# Discovery of neuroprotective soluble guanylate cyclase modulators (GCMs) aciting via NO/GC/cGMP/CREB pathway

**DOI:** 10.1186/2050-6511-14-S1-P65

**Published:** 2013-08-29

**Authors:** Marton I Siklos, Ronak P Gandhi, Rui Xiong, Isaac T Schiefer, Vladislav Litosh, Lawren VandeVrede, Gregory RJ Thatcher

**Affiliations:** 1 Department of Medicinal Chemistry & Pharmacognosy, University of Illinois College of Pharmacy, University of Illinois at Chicago (UIC), Chicago, IL 60612, USA

## 

NO/cGMP signaling is essential for normal brain function, including learning and memory, and mediation of long-term potentiation (LTP). NO/cGMP signaling is coupled to cholinergic, glutamatergic, and dopaminergic systems and plays key roles in motor function associated with Parkinson’s disease pathogenesis and L-DOPA therapy. In Alzheimer’s Disease (AD), early synaptic failure, has been linked to dysfunction of gene expression programs mediated via the transcription factor cAMP-response element binding protein (CREB), activation of which is tightly regulated by NO/cGMP. Since several neurodegenerative disorders continue to be dogmatically linked to the chemical toxicity of NO, activation of soluble guanylyl cyclase (sGC) represents a potential therapeutic approach in both AD and PD; whereas negative modulation of sGC may be of use in some stages of PD. Such small molecule sGC modulators (GCMs) can be assayed in cell cultures by measuring levels of phosphorylated CREB (pCREB). GCMs that allosterically potentiate NO activation of sGC have proven successful in clinical trials for peripheral indications, however, there are no reports directed at therapeutic activity in the CNS. The aim of this study is to develop GCMs for use in CNS disorders, including AD and PD. A library of GCMs was synthesized, including positive allosteric modulators (PAMs) and negative modulators, as assessed by increasing levels of pCREB in SH-SY5Y human neuroblastoma cell cultures. Molecules were designed by classical bioisosteric replacement, aiming for desirable physiochemical properties and chemical diversity. GCM PAMs were assayed for cGMP elevation and reversal of neurotoxicity induced by 6-hydroxydopamine in dopaminergic neuronal cells. One GCM PAM, active in cell culture, was further tested for the reversal of memory deficits in mice treated with scopolamine, and drug levels in brain and plasma measured. The ligand binding site for sGC stimulators and GCM PAMs on the sGC protein remains to be definitively defined. Photoaffinity probes were designed that retained the activity of the parent GCM PAM in cell cultures in order to elucidate the binding mode to sGC and to indicate other protein partners for such compounds.

